# Aging time influences fatty acid profiles and volatile compounds in cooked Thai native beef

**DOI:** 10.5455/javar.2025.l885

**Published:** 2025-03-25

**Authors:** Watcharawit Meenongyai, Kunwadee Kaewka, Kannika Wongpanit, Piyamas Phongkaew, Pichad Khejornsart, Panuwat Khumpeerawat, Alexander Michael Stelzleni

**Affiliations:** 1 Department of Agriculture and Resources, Faculty of Natural Resources and Agro-Industry, Kasetsart University Chalermphrakiat Sakon Nakhon Province Campus, Sakon Nakhon, Thailand; 2 Department of Food Technology and Nutrition, Faculty of Natural Resources and Agro-Industry, Kasetsart University Chalermphrakiat Sakon Nakhon Province Campus, Sakon Nakhon, Thailand; 3 Department of Animal and Dairy Sciences, University of Georgia, Athens, GA, USA

**Keywords:** Beef, dry aged, fatty acid profiles, flavor, volatile compounds

## Abstract

**Objective:**

This study aims to assess the impact of aging time on the quality of meat, fatty acid profiles, and volatile compounds in cooked Thai native beef.

**Materials and Methods:**

The experiment utilized a randomized complete block design, where the aging time (0, 7, 14, 21, and 28 days) served as the treatment and the longissimus thoracis et lumborum muscle from Thai native cattle (*n* = 4) was considered a block.

**Results:**

Meat color and Warner-Bratzler shear force values decreased as the aging time increased. Furthermore, the moisture content decreased while the protein and ash content increased with a longer aging time. The proportions of fatty acids showed significant differences with varying aging times. These fatty acids exhibited the highest proportions in the 14-day dry-aged beef samples. The levels of short-chain aldehydes (pentanal, heptanal, octanal, nonanal, decanal, and 2-nonenal) and alcohols (1-hexanol and 1-octanol) decreased as the aging time increased. However, there was an increase in long-chain aldehydes (tetradecanal, hexadecanal, heptadecanal, and octadecanal) with longer aging time. In addition, the levels of toluene, dodecane, tridecane, methyl-pyrazine, 2,5-dimethyl-pyrazine, trimethyl-pyrazine, and dimethyl trisulfide were higher in 14-day dry-aged beef compared to beef that had not been aged. Furthermore, a correlation was found between the number of the 6 significant fatty acids in the dry-aged beef samples and the 12 volatile compounds in cooked beef.

**Conclusion:**

Our results suggest that aging times significantly influence the fatty acid profiles of Thai native beef, which in turn are correlated with the characteristic volatile compounds.

## Introduction

Thai native cattle are a small breed of *Bos indicus* type used mainly for beef production in Thailand. Mature males typically weigh between 300 and 350 kg, while females average 200 to 270 kg [1]. Despite its smaller size, Thai native beef is sought after by segments of the population due to its higher proportion of polyunsaturated fatty acids (PUFAs) and conjugated linoleic acid (CLA), offering a leaner alternative with less fat and cholesterol compared to beef from *Bos taurus* breeds [2]. However, the larger muscle fibers and greater connective tissue in *B. indicus* beef can result in less tenderness [3].

Postmortem aging, particularly dry aging, is a well-established method to enhance the tenderness and overall palatability of beef [4]. Dry aging can also influence the flavor profile by altering the volatile compounds through lipid oxidation and the Maillard reaction [5]. Common aromatic compounds in cooked meat include short-chain aliphatic aldehydes with C6-C10 carbons, 1-octen-3-ol (or 1-octen-3-one), and heterocyclic compounds with sulfur or nitrogen [6]. The flavor of cooked meat may depend on the concentration and odor threshold of these compounds, with the reaction products (pyrazines, pyrroles, oxazoles, and thiophenes) generally described as strong odorants [5]. The specific breed of animal within a species has been reported as a contributing factor influencing the volatile compounds of cooked meat [7].

While the effects of aging on beef characteristics and volatile compounds in *B. taurus* are well-documented [4–7], there is limited research on the influence of aging duration on the volatile compound profiles of *B. indicus*, particularly Thai native beef, and their potential association with fatty acid changes. Given that Thai native cattle have a higher proportion of PUFA compared to *B. taurus* cattle [8], coupled with the lipid oxidation that occurs during dry aging and the Maillard reaction of cooked dry-aged beef [7], it is hypothesized that extended exposure to dry aging (0, 7, 14, 21, and 28 days) would impact the fatty acid profiles of Thai native beef, leading to differences in volatile compounds.

Therefore, the objective of this study was to quantify the effects of varying dry aging times on the fatty acid composition and volatile compound profile of Thai native beef. By exploring these relationships, the study aims to provide novel insights into the mechanisms underpinning flavor and quality development in *B. indicus* beef, thereby contributing to the global understanding of breed-specific aging impacts.

## Materials and Methods

### Ethical approval

The experimental procedures received approval from the Institutional Animal Care and Use Committee at Kasetsart University, Thailand (Approval No. ACKU66-CSC-004).

### Animal handling and beef sample preparation

The longissimus thoracis et lumborum (LTL) muscles were chosen from four Thai native cattle (both left and right sides) at a commercial slaughterhouse located in Sakon Nakhon, Thailand. These four Thai native cattle bulls, all 3 years old, were raised on the same commercial farm under feedlot conditions for 4 months. Throughout this time, they were consistently provided with the same diet. The LTL samples were transported to the Meat Science Laboratory at Kasetsart University, Thailand, 2 h after collection, maintaining cold conditions in an icebox. Starting at the second rib the LTL was fabricated into 5 sections 10.0 cm thick. Sections were then randomly assigned to 0, 7, 14, 21, and 28 additional days of dry aging at 4°C with a relative humidity of 80% and an airflow velocity of 3 m/s. The left side of the LTL muscle was used for color, chemical composition, and fatty acid profile analysis. The right LTL was used for Warner-Bratzler shear force (WBSF) and volatile compound evaluation. After the assigned day of aging, the respective sections were trimmed of fat and connective tissue, and the steak was cut 1.5 cm thick. Each steak was individually vacuum-sealed using a PA/PE bag (20 × 30 cm, 180 µm thick, Spring Green Evolution Co. Ltd., Bangkok, Thailand) and subsequently stored at −20°C until analysis.

### Meat color and proximate analysis

Objective steak surface color (L*, a*, b*) was measured on the uncovered LTL steak after approximately 30 min of bloom at 4°C (Minolta CR-300, Minolta Inc., Osaka, Japan; illuminant D65, 2° view angle, and 50 mm diameter calibrated to a standard white tile following equipment instructions) by taking the average of 6 scans. Meat samples for proximate analysis were thawed (4°C, 12 h), had visible external fat and connective tissue removed, and the separable lean from each sample was hand chopped and thoroughly mixed. Moisture, protein, fat, and ash were determined following the AOAC method [9] in triplicate.

### WBSF determination

The steaks were taken out of the freezer and left to thaw at 4°C for 24 h. Subsequently, beef samples were cooked sous vide at the same time in a preheated water bath (Memmert Waterbath WB7, Schwabach, Germany) at 80°C for 75 min. All of the samples were cooled in ambient running tap water for 40 min after removal from the water bath to bring them to room temperature (25°C). All steak was removed from the vacuum bag, trimmed, and cut into 1.27 cm diameter cores. The shear force of the cooked beef samples was measured using the Warner-Bratzler blade of the TA-XT2i texture analyzer (Texture Technologies Corp. and Stable Micro Systems, Surrey, UK), equipped with a 5 kN load cell and operated at a crosshead speed of 200 mm/min.

### Fatty acid profile analysis

Lipids for fatty acid analysis were extracted by the chloroform-methanol method. Briefly, 90 ml of chloroform: methanol in a 2:1 ratio, along with 25 mg of butylated hydroxyanisole (BHA, 10%) dissolved in 98% ethanol, were introduced to 15 gm of the sample within a 250-ml Erlenmeyer flask and homogenized at low speed for 2 min using a Nissei AM-8 Homogenizer (Nihon Seiki Co., LTD., Osaka, Japan). The blended sample was strained through a No. 1 Whatman filter paper into a 250-ml separatory funnel. Subsequently, a solution comprising 30 ml of chloroform, 30 ml of deionized water, and 50 ml of 0.58% NaCl was added. Once the separatory funnel was sealed with a glass stopper, the filtrate was thoroughly mixed, allowing the contents to settle until the aqueous and organic layers were distinctly separated. The aqueous layer was discarded, and the organic lipid layer was then transferred to an Erlenmeyer flask. Sodium sulfate (Na_2_SO_4_) in a 1:1 weight/volume ratio was added to the flask to facilitate further dehydration of the sample. The lipid fraction was subsequently filtered into a round-bottom flask using Whatman paper No. 1 and evaporated at 40°C utilizing a rotary evaporator (BUCHI Rotavapor R-200, BUCHI Labortechnik AG, Flawil, Switzerland). The remaining lipid was transferred to amber vials, purged with nitrogen gas (N_2_), sealed, and kept at −20°C until methylation.

Methylation procedures were conducted by adding 25 mg of lipid to a screw-cap culture tube, followed by the addition of 15 ml of NaOH/MeOH (0.5 N) and 1.5 ml of nitrogen-purged methanol. The tube was sealed, purged with nitrogen gas, and incubated in a water bath at 100°C for 5 min. After reaching the ambient temperature, 2 ml of a 14% BF_3_/MeOH solution was introduced while under a blanket of nitrogen gas, stirred completely, and then heated in a water bath at 100°C for 5 min. Once cooled back to room temperature, 10 ml of deionized water was added, and the mixture was transferred to a 50 ml centrifuge tube. Finally, the tube was rotated at 2,800 × *g* for 15 min at 10°C to separate the phases. The hexane layer containing the methylated fatty acids was used for gas chromatographic analysis (GC, Agilent, 7890a) and equipped with an automatic sample injector and a flame ionization detector. A flexible fused silica capillary column (SPTM-2560; 0.25 mm i.d., 100 m, 0.20 μm film thickness; Supelco Inc., Bellefonte, PA, USA) was used. The sample was divided into a capillary column (1 μl and portioning ratio 10:1 μl). The heating cycle began at 70°C for 4 min, an increase of 13°C/min to 175°C and keeping this temperature for 27 min, followed by a rise of 4°C/min to 215°C and a 17 min hold, then an increase to 240°C at 4°C/min, and a final 10 min keep. Finally, the inlet and detector temperatures were 240°C and 280°C, respectively. Helium was used as the carrier gas, and a constant column flow rate of 0.9 ml/min was used. The flow rates of air, hydrogen (H_2_), and makeup gas (N_2_) in the flame ionization detector were 400, 30, and 25 ml/min, respectively. Fatty acids were identified using the retention times of known commercial standards (Supelco 37 Comp. FAME Mix, Supelco Inc; catalog number: 1245. Matreya LLC, State College, PA, USA) and expressed as a percentage of total fatty acids.

### Meat cooking preparation

All steaks were thawed at 4°C for 24 h. After steak samples reached 2°C–5°C they were stored at room temperature for 2 h. Steaks were cooked using an electric air fryer (CO-745, OTTO, Thailand) that was preheated for 5 min to 200°C. The steaks were cooked in the air fryer at 200°C for 6 min until reaching a core temperature of 71°C ± 1°C. After cooking, the steaks were cooled to room temperature (25°C), minced, vacuum packaged, and frozen at −80°C in preparation for volatile analysis.

### Extraction of volatiles

The minced meat samples were defrosted for 2 h at room temperature (25°C). Approximately 10 gm of the cooked sample was weighed into a 20 ml crimp vial. The vials were sealed with a PTFE septum and steel magnetic cap (Agilent Technologies). Sampling was done via solid-phase microextraction (SPME) on a 30/50 μm DVB/Carboxen/PDMS fiber (Supelco) along with the G6509B GC sampler 120 autosampler (Agilent Technologies, USA). The extraction process was completed over 40 min at 80°C with 15 min of pre-equilibration time at the extraction temperature.

### Gas chromatography-mass spectrometry conditions

A 7890A GC and 7000B MS detector (Agilent Technologies) were used to analyze volatile compounds. Injector port desorption of volatiles on the SPME-fiber took place in split mode (split ratio 5:1) within 5 min at 240°C. A DB-wax capillary column (length of 60 m, inner diameter of 0.25 mm, film thickness of 0.25 μm, Agilent Technologies) was used to separate the volatiles. The column operated alongside this temperature gradient: maintaining an oven temperature of 40°C for 5 min, then rising by 5°C every min until reaching 110°C, then rising by 8°C/min until reaching 240°C, and finally maintaining the last temperature for 15 min. The helium of 99.999% purity with a constant pressure of 206.84 kPa was employed as the carrier gas to convey the volatiles to the MS detector through a transfer line maintained at 240°C. An initial identification was achieved by comparing the experimental spectra with the Mass Spectral Library of the National Institute of Standards and Technology. Peak area unit (AU) values of 106/gm are used to represent data.

### Statistical analysis

The experiment utilized a randomized complete block design, where the aging time (0, 7, 14, 21, and 28 days) served as the treatment, and the LTL muscle from Thai native cattle (*n* = 4) was considered a block. The effects of different aging times on the chemical compositions, physical characteristics, fatty acid profiles, and volatile compounds of the meat were analyzed using PROC ANOVA of SAS version 9.0 (SAS Institute Inc., Cary, NC). Mean comparisons were conducted using Duncan’s New Multiple Range Test, and differences were deemed significant when *p* < 0.05. Tendencies were considered when 0.05 ≤ *p* ≤ 0.10. The correlation data were analyzed using SPSS 17.0 (IBM Corporation, Armonk, NY).

## Results and Discussion

### Effect of aging time on the instrumental color, WBSF, and chemical composition of Thai native beef

The effects of aging time on the meat color, WBSF, and chemical composition of Thai native beef are presented in Table 1. The observed changes in color parameters, including lightness (L*), redness (a*), and yellowness (b*), highlight the dynamic biochemical processes occurring during aging. The darker appearance (lower L*) of the LTL muscle after 7, 21, and 28 days of aging, compared to 0 and 14 days (*p* < 0.01), reflects oxidative changes, and potential moisture loss [10]. Redness (a*) decreased significantly after 7 days, while yellowness (b*) progressively declined with extended aging (*p* < 0.01). These findings align with the hypothesis that prolonged aging increases lipid oxidation and promotes the formation of metmyoglobin, a pigment associated with discoloration in meat [4].

The decline in WBSF values with increasing aging time (*p* < 0.01) highlights the tenderizing effects of proteolysis. The lowest WBSF values were observed at 21 and 28 days, suggesting that this period maximizes tenderness improvements. The action of calpains, particularly μ-calpain, is well-established as a key factor in postmortem proteolysis, targeting myofibrillar and cytoskeletal proteins [11]. These enzymatic activities degrade structural proteins such as desmin and titin, contributing to the breakdown of the muscle matrix and improving tenderness [12]. Our findings corroborate those of Bhat et al. [13], who reported a 17% reduction in shear force from day 14 to day 35 of dry aging. Although prolonged aging enhances tenderness, oxidative changes may adversely affect consumer perception. Further research should investigate interventions such as vacuum packaging or antioxidant supplementation to optimize the sensory and nutritional quality of aged Thai native beef.

Moisture content was greatest after 0 days of aging (*p <* 0.01; Table 1). Unexpectedly, samples aged for 7 days exhibited lower moisture content compared to those aged for 0, 21, and 28 days (*p* < 0.01), while samples aged for 21 and 28 days showed similar moisture levels (*p* = 0.0008). This could be a possibility, even if the placement is random. However, it is worth noting that the samples dry-aged for 7 days were situated in areas that had greater exposure to airflow. Overall, these results were in agreement with those of Passetti et al. [8], who reported that the moisture content of beef decreased as dry aging time increased from 0 to 28 days.

However, the constant moisture content after the 14-day dry aging might result from the development of a protective crust on the meat surface. This crust acts as a barrier, preventing excessive moisture loss and retaining moisture within the product [14]. Counter to moisture, dry-aging increased in the proportion of protein and ash as time increased (*p* < 0.01). As the proportion of moisture decreased due to losses to the ambient environment, it was expected that the proportion of other components, including protein, would increase [15]. The proportion of lipids did not differ across dry aging times (*p = *0.94). This finding was consistent with those of Fan et al. [16], who found that the fat content in the dry-aged beef showed no differences between aging days and can partially be explained by the low lipid content of the samples. These findings reveal the impact of environmental conditions on dry aging and meat quality. Future research should focus on standardizing airflow and exploring the protective crust as a natural barrier to minimize dehydration and reduce artificial interventions.

**Table 1.** Means of the physicochemical properties and chemical composition of dry-aged Thai beef dry aged for 28 days.

Item	Day of aging (days)					SEM	*p-*value
	0	7	14	21	28		
Color							
L*	34.19^a^	31.74^b^	35.21^a^	30.49^b^	31.38^b^	0.36	< 0.01
a*	11.32^a^	12.24^a^	9.79^b^	8.69^b^	9.14^b^	0.23	< 0.01
b*	11.94^a^	10.65^ab^	11.67^a^	9.40^b^	8.76^b^	0.30	< 0.01
WBSF (N)	123.40^a^	83.59^b^	93.40^b^	55.17^c^	60.59^c^	3.58	< 0.01
Moisture, (%)	70.98^a^	64.41^c^	65.29^bc^	67.65^b^	67.70^b^	0.57	< 0.01
Protein, (%)	19.70^c^	27.77^a^	26.99^a^	25.27^ab^	23.39^b^	0.66	< 0.01
Lipid, (%)	1.46	1.21	1.32	1.36	1.40	0.10	0.94
Ash, (%)	3.74^d^	4.29^dc^	4.61^bc^	4.95^ab^	5.51^a^	0.13	< 0.01

^a,b,c,d ^Means with different superscripts within the same row are statistically significant differences (*p* < 0.05). L*: Lightness, a*: Redness, b*: Yellowness. SEM = standard error of the mean.

### Effect of dry aging time on the fatty acid profiles of Thai native beef

The fatty acid composition of Thai native beef dry aged for 0 to 28 days is shown in Table 2. The percentage of gamma-linolenic acid (C18:3n6) was greater after 14 days of aging than at 0 and 28 days (*p* < 0.05). Eicosapentaenoic acid (C20:5n3), behenic acid (C22:0), and docosahexaenoic acid (C22:6n3) were greatest after 14 days (*p* < 0.01) than all other days. Lignoceric acid (C24:0) was greater after 14 days than at 0, 21, and 28 days of aging (*p* < 0.05). These results highlight a distinct change in fatty acid profiles during the mid-phase of dry aging. In addition, total *ω*-3 fatty acid (n-3) content was also greatest after 14 days (*p* < 0.01), resulting in lower *ω*-6:* ω*-3 (n-6/n-3) ratios in 14-day dry-aged Thai native beef than in 7 and 28 days dry-aged beef (*p* < 0.05). This finding is particularly noteworthy, as a lower n-6/n-3 ratio is associated with enhanced health benefits [17], making 14-day dry-aged beef potentially more desirable from a nutritional perspective.

**Table 2.** Means for fatty acid profiles of Thai beef dry aged for 28 days.

Item	Day of aging (days)					SEM	*p-*value
	0	7	14	21	28		
	-------------------- gm /100 gm ----------------------						
C10:0	0.04	0.03	0.03	0.04	0.04	<0.01	0.32
C12:0	0.30	0.28	0.13	0.31	0.36	0.04	0.48
C13:0	0.05^x^	0.03^y^	0.04^y^	0.03^y^	0.04^y^	<0.01	0.05
C14:0	4.33	3.56	3.03	3.99	4.51	0.25	0.41
C14:1	1.13	0.73	0.63	0.78	0.85	0.07	0.14
C15:0	0.43	0.46	0.47	0.44	0.48	0.02	0.95
C16:0	26.58	23.94	23.55	25.84	26.96	0.64	0.43
C16:1	4.41^x^	3.52^y^	3.58^y^	3.64^y^	3.90^xy^	0.12	0.09
C18:0	13.63	16.78	14.79	16.24	15.86	0.40	0.11
C18:1n9c	39.41	37.47	37.64	38.15	35.71	0.56	0.36
C18:2n6c	3.15	4.70	5.31	3.67	3.74	0.30	0.23
C18:2 c9, t11	1.89^x^	1.14^y^	1.07^y^	1.54^xy^	2.07^x^	0.14	0.08
C18:2 c9, c11	0.26	0.40	0.41	0.35	0.28	0.04	0.64
C18:2 t9,t11	0.39	0.32	0.40	0.34	0.27	0.03	0.65
C18:2 t10, t12	0.21	0.26	0.20	0.22	0.29	0.01	0.17
C18:3n3	0.50	0.45	0.59	0.43	0.41	0.03	0.21
C18:3n6	0.03^b^	0.06^ab^	0.09^a^	0.05^ab^	0.04^b^	0.01	0.04
C20:1n9	0.10	0.15	0.13	0.14	0.13	<0.00	0.37
C20:2n6	0.04	0.08	0.07	0.07	0.07	0.01	0.68
C20:3n6	0.35	0.79	0.88	0.58	0.67	0.07	0.18
C20:4n6	1.47	3.09	3.75	2.03	2.20	0.30	0.17
C20:5n3	0.09^b^	0.11^b^	0.25^a^	0.10^b^	0.08^b^	0.02	< 0.01
C22:0	0.10^b^	0.07^b^	0.14^a^	0.08^b^	0.08^b^	0.01	< 0.01
C22:6n3	0.25^b^	0.44^b^	0.95^a^	0.27^b^	0.26^b^	0.08	< 0.01
C24:0	0.71^b^	0.95^ab^	1.69^a^	0.46^b^	0.50^b^	0.14	0.02
SFA	46.29	46.27	44.05	47.64	49.02	0.95	0.66
MUFA	45.04	41.86	41.97	42.70	40.59	0.58	0.15
PUFA	8.66	11.86	13.97	9.65	10.39	0.72	0.23
n-6	5.00	8.64	10.02	6.32	6.66	0.66	0.18
n-3	0.86^b^	1.01^b^	1.79^a^	0.80^b^	0.77^b^	0.11	0.01
n-6/n-3	6.45^bc^	9.02^a^	5.70^c^	7.78^abc^	8.28^ab^	0.44	0.04
Total CLA	2.74	2.12	2.09	2.45	2.89	0.12	0.15

^a,b,c ^Means with different superscripts within the same row are statistically significant differences (*p* < 0.05). ^x,y,z ^Means with different superscripts within the same row are statistically significant differences (*p* < 0.1). SFA = saturated fatty acid, MUFA = monounsaturated fatty acid, PUFA = polyunsaturated fatty acid, n-3 = omega-3 fatty acid, n-6 = omega-6 fatty acid, CLA = conjugated linoleic acid.

The observed increase in C18:3n6, C20:5n3, C22:6n3, and total n-3 content at 14 days of aging, followed by a decline, suggests dynamic biochemical changes during the aging process. While lipid oxidation during aging is well-documented as a driver of unsaturated fatty acid degradation [6,7], the transient increase observed in this study is less understood. One possible explanation could be enzymatic hydrolysis or the release of bound fatty acids during the early to mid-aging period [7]. Additional studies are required to elucidate the mechanisms underlying these changes, particularly in Thai native beef, which may exhibit unique lipid metabolism due to its genetic and environmental factors. Tridecanoic acid (C13:0) tended to be greater after 0 days than 7, 21, and 28 days (*p* = 0.05) and palmitoleic acid (C16:1) tended to be greater after 0 days than 7, 14, and 21 days (*p *= 0.09). It has been established that cis fatty acids are more prone to oxidation than trans fatty acids, which implies cis fats could have decayed more substantially during the aging process [18]. CLA, C18:2 c9,t11 tended to be greater after 0 days than 7 and 14 days (*p* = 0.08). The stability of CLA during aging, despite lipid oxidation, further supports its relative resistance due to its chemical structure, which lacks methylene-interrupted double bonds [19]. Proportions of all other fatty acids did not differ due to dry aging time (*p* > 0.10) resulting in no differences (*p* > 0.10) for total fatty acid classification of monounsaturated fatty acid (MUFA), PUFA, and saturated fatty acid (SFA). The increase in beneficial fatty acids at 14 days suggests opportunities to optimize aging for improved nutritional value and consumer appeal. Future research should focus on the enzymatic and oxidative pathways driving these changes.

### Effect of dry aging time on the volatile compounds of cooked Thai native beef

A total of 38 volatile compounds were identified including 17 aldehydes, 1 ketone, 5 alcohols, 2 acids, 1 aromatic hydrocarbon, 3 aliphatic hydrocarbons, 7 nitrogen- and oxygen-containing heterocyclic compounds, 1 sulfur-containing compound, and 1 ester compound (Tables 3 and 4). Cooked meat contains volatiles such as aldehydes, alcohols, aliphatic hydrocarbons, ketones, and esters that are produced, at least partially, from the oxidation of fatty acids [6]. Additionally, sulfur- and nitrogen-containing heterocyclic compounds originating from water-soluble precursors via the Maillard reaction have substantially lower odor detection thresholds than those for lipid-derived compounds [7].

### Aldehydes

In this study, aldehydes were the predominant group of volatile compounds identified in cooked Thai native beef, a finding that aligns with the work of Sohail et al. [6], who also identified aldehydes as significant contributors to the aroma of cooked meat. The high concentration of aldehydes in cooked beef may be attributed to the lipid content and the thermal oxidation of fatty acids that occur during cooking [7]. Additionally, this study observed that dry aging significantly influenced the aldehyde profile of Thai native beef, highlighting the role of lipid oxidation in flavor development [20]. Specifically, 10 aldehyde compounds were significantly affected by dry aging time (*p* < 0.05), including pentanal, heptanal, octanal, nonanal, decanal, and 2-nonenal, which were more concentrated in the 0-day samples compared to those aged for longer periods (Table 3). This result suggests that shorter aging periods may result in higher concentrations of these specific aldehydes, likely due to a faster rate of oxidation early in the aging process. Conversely, tetradecanal, hexadecanal, heptadecanal, and octadecanal were found to be more concentrated in samples aged 14, 21, and 28 days compared to the 0-day samples (*p* < 0.05). These changes indicate that prolonged dry aging may enhance the production of certain aldehydes, which could be a result of extended lipid oxidation [20].

A key observation in this study is that the concentrations of aldehydes such as tetradecanal, hexadecanal, heptadecanal, and octadecanal increased with extended aging time, while the total aldehyde content did not differ significantly between aging times (*p* = 0.14). This finding contrasts with the study by Lee et al. [20], which reported an increase in total aldehyde content due to lipid oxidation during dry aging. The lack of a significant increase in total aldehyde content in our study could suggest that while lipid oxidation continues during the dry aging process, other factors such as the development of a protective crust [14] or the depletion of oxidative substrates after prolonged aging might limit the accumulation of aldehydes overall [7].

### Ketone

The only ketone found was 2-butanone (Table 3). This suggests that a specific point in the dry aging process may trigger a peak in 2-butanone formation, likely related to the interaction of oxidative processes and the composition of fatty acids in Thai native beef. Ketones are generally recognized as lipid-oxidation products with low odor detection thresholds [20]. In comparison to other lipid-derived aroma compounds such as aldehydes or esters, most ketones typically have a higher odor threshold [21]. The 2-butanone content in dry-aged beef fluctuated as the aging duration increased and was greater after 0 and 14 days of aging than at any other time (*p* < 0.01). 2-Butanone is formed by the oxidation of fatty acids and is commonly found in cooked beef [22]. It has been suggested that increased 2-butanone levels in cooked beef positively affect flavor and aroma [23]. While 2-butanone is recognized as an important flavor compound, its concentration and formation timing during dry aging appear to be influenced by factors specific to Thai native beef. Further research is needed to clarify the mechanisms behind the temporal fluctuations of 2-butanone and examine how aging conditions such as humidity, temperature, and airflow affect its production.

**Table 3.** Mean for the peak area of aldehydes, ketones, and alcohol (AU ×10^6^/gm) from cooked Thai native beef dry aged for 28 days.

Volatile compounds	Day of aging (days)					SEM	*p-*value
	0	7	14	21	28		
Aldehydes							
Acetaldehyde	9.96^y^	7.39^z^	11.04^x^	8.07^z^	7.93^z^	0.48	0.06
Pentanal	2.64^a^	1.81^b^	0.00^c^	0.00^c^	0.00^c^	0.30	< 0.01
Hexanal	31.51^x^	11.45^y^	9.55^y^	7.66^y^	5.79^y^	3.07	0.05
Heptanal	16.83^a^	7.21^b^	6.13^b^	7.55^b^	7.07^b^	0.96	< 0.01
Octanal	40.44^a^	15.20^c^	13.30^c^	23.42^bc^	29.26^b^	2.71	< 0.01
Nonanal	215.36^a^	89.28^c^	94.14^c^	154.04^ab^	154.33^ab^	14.16	< 0.01
Decanal	5.14^a^	3.62^b^	3.58^b^	3.39^bc^	2.36^c^	0.25	< 0.01
2-Nonenal	3.56^a^	1.27^c^	1.07^c^	1.63^bc^	2.50^b^	0.25	< 0.01
Dodecanal	3.21^x^	2.11^y^	3.26^x^	2.61^xy^	2.30^xy^	0.17	0.07
Tridecanal	3.63^x^	2.22^y^	3.08^xy^	2.12^y^	2.17^y^	0.21	0.07
Tetradecanal	7.10^b^	7.41^b^	13.33^a^	8.21^b^	9.50^b^	0.72	0.04
Pentadecanal	15.35^y^	17.22^y^	28.69^x^	19.20^y^	19.26^y^	1.52	0.09
Hexadecanal	72.95^c^	132.03^bc^	215.19^a^	154.27^ab^	178.96^ab^	13.49	< 0.01
Heptadecanal	2.67^b^	3.54^b^	6.35^b^	7.44^b^	34.28^a^	1.10	0.04
Octadecanal	6.16^b^	10.28^ab^	17.80^ab^	12.91^ab^	21.07^a^	1.37	< 0.01
9-Octadecenal, (Z)-	1.81^y^	4.09^xy^	7.04^x^	3.59^xy^	8.03^x^	2.30	0.07
Benzaldehyde	156.08^x^	102.81^xy^	151.07^x^	93.72^z^	113.10^xy^	8.89	0.06
Total aldehydes	594.39	418.93	584.62	509.82	587.91	25.94	0.14
Ketone							
2-Butanone	3.85^a^	2.55^b^	3.45^a^	2.50^b^	2.58^b^	0.16	< 0.01
Alcohols							
1-Hexanol	1.70^a^	1.45^b^	0.00^c^	0.00^c^	0.00^c^	0.18	< 0.01
1-Octen-3-ol	4.64	3.68	5.82	3.76	2.79	0.50	0.50
1-Octanol	18.60^a^	8.96^b^	8.65^b^	13.71^ab^	14.21^ab^	1.16	0.01
Trans-2-Dodecen-1-ol	7.06	4.27	7.91	4.78	4.07	0.63	0.12
1-Dodecanol	0.00^c^	0.00^c^	0.00^c^	2.88^b^	4.97^a^	0.50	< 0.01
Total alcohols	32.00^a^	18.37^b^	22.39^b^	25.13^ab^	26.04^ab^	1.47	0.03

^a,b,c ^Means with different superscripts within the same row are statistically significant differences (*p* < 0.05). ^x,y,z ^Means with different superscripts within the same row are statistically significant differences (*p* < 0.1). SEM = standard error of the mean.

### Alcohols

The total alcohol content in dry-aged Thai native beef exhibited fluctuations as aging time progressed (Table 3). Notably, 1-hexanol concentrations decreased with extended aging, while 1-octanol showed a marked decline from 0 to 7 days and remained stable throughout the remainder of the aging period (*p* ≤ 0.01). Conversely, 1-dodecanol increased significantly after 21 days of aging and continued to rise through 28 days (*p* < 0.01). These findings are partially consistent with the results of previous studies, such as that of Sohail et al. [6], who reported a significant increase in the straight-chain alcohol content in dry-aged beef as aging time increased. The observed trend was attributed to the oxidation of unsaturated fatty acids. However, the present study observed a decline in alcohol content during the extended aging period, which warrants further investigation. While lipid oxidation is expected to produce alcohols like 1-hexanol and 1-octanol, the reasons behind the specific decline in alcohol concentrations during extended aging in this study remain unclear. Further research is necessary to explore the mechanisms underlying these fluctuations. In contrast, no significant differences were observed in branched-chain alcohols such as 1-octen-3-ol and trans-2-dodecen-1-ol (*p* ≥ 0.10). This suggests that the production of branched-chain alcohols remained relatively stable throughout the aging process, indicating consistent microbial activity across the different aging periods. This aligns with the findings of Sharma et al. [24], who noted that branched-chain alcohols, particularly those with low molecular weights, are produced primarily through microbial fermentation in meat.

### Acids and esters

The concentrations of acetic acid and dodecanoic acid in dry-aged Thai native beef remained consistent across all aging periods (*p* > 0.05; Table 4). Acids are crucial precursors in the esterification process, contributing significantly to the aroma profiles of beef products by serving as substrates for ester formation [21]. The stable acid levels observed across aging periods suggest that metabolic processes influencing acid production and degradation, such as lipid oxidation or microbial fermentation, remained unaffected by the duration of dry aging [22]. Similarly, the ester 1-propen-2-ol acetate was the sole ester compound identified in this study, and its concentration did not differ significantly among the aging periods (*p* > 0.05). Esterification of alcohols and acids leads to the formation of esters, which is associated with the activity of microbial esterase [25]. The stable ester content observed across all aging times suggests that microbial metabolic activity related to ester formation, as well as the esterification process itself, remained consistent during the dry-aging period [21]. Interestingly, the lack of variation in both acid and ester concentrations contrasts with other volatile compounds, such as aldehydes and alcohols, which were more responsive to aging duration. Previous studies, such as those by Li et al. [26], highlighted that microbial activity often varies with aging conditions, influencing ester production. However, the results of this study imply that the specific conditions of dry aging employed, potentially including controlled temperature, humidity, and airflow, maintained a stable microbial environment, leading to uniform ester formation over time.

**Table 4.** Mean for the peak area of acids, ester, aromatic- and aliphatic-hydrocarbons, nitrogen- and oxygen-containing heterocyclic compounds, and sulfur-containing compounds (AU ×10^6^/gm) from grilled dry-aged beef with different aging times.

Volatile compounds	Day of aging (days)					SEM	*p-*value
	0	7	14	21	28		
Acids							
Acetic acid	0.77	0.97	1.84	3.70	12.05	1.67	0.18
Dodecanoic acid	2.22	3.46	1.86	1.45	1.85	0.84	0.10
Total acids	2.99	4.44	3.70	5.15	13.90	1.74	0.28
Ester							
1-Propen-2-ol, acetate	6.75	5.99	8.78	6.45	8.36	0.56	0.39
Aromatic hydrocarbons							
Toluene	2.42^b^	2.56^b^	3.96^a^	2.33^b^	1.77^b^	0.22	0.01
Aliphatic hydrocarbons							
Dodecane	1.80^b^	3.01^b^	6.37^a^	4.96^a^	3.23^b^	0.41	< 0.01
Tridecane	2.75^b^	2.73^b^	8.24^a^	2.86^b^	2.28^b^	0.54	< 0.01
Hexadecane	7.49^ab^	5.00^b^	7.54^ab^	11.55^a^	9.98^a^	0.75	0.04
Total aliphatic hydrocarbons	12.04^c^	10.75^c^	22.15^a^	19.36^ab^	15.50^bc^	1.20	< 0.01
Nitrogen-containing heterocyclic compounds							
Methyl-Pyrazine	0.00^c^	0.00^c^	2.29^a^	0.89^b^	1.47^ab^	0.24	<0.01
2,5-dimethyl-Pyrazine	1.61^b^	1.87^b^	5.93^a^	1.17^b^	2.66^b^	0.55	0.04
Trimethyl-Pyrazine	0.00^c^	0.00^c^	24.67^a^	30.35^a^	23.37^a^	3.12	<0.01
3-ethyl-2,5-dimethyl-Pyrazine	1.82	3.15	8.05	3.45	4.18	0.76	0.11
2,6-diethyl-Pyrazine	0.00^b^	1.00^a^	0.00^b^	0.00^b^	0.00^b^	0.11	<0.01
3-Acetyl-1H-pyrroline	2.31^xy^	1.44^y^	3.15^x^	1.63^xy^	2.94^xy^	0.24	0.09
Total nitrogen-containing heterocyclic compounds	5.74^b^	7.46^b^	44.09^a^	37.51^a^	34.63^a^	3.99	<0.01
Oxygen-containing heterocyclic compounds							
Furan, 2-pentyl-	5.27	3.14	4.22	2.30	1.84	0.51	0.27
Sulfur-containing compound							
Dimethyl trisulfide	3.75^ab^	2.55^bc^	4.59^a^	2.30^c^	3.16^bc^	0.26	0.03

^a,b,c ^Means with different superscripts within the same row are statistically significant differences (*p* < 0.05). ^x,y,z ^Means with different superscripts within the same row are statistically significant differences (*p* < 0.1). SEM = standard error of the mean.

### Aromatic- and aliphatic-hydrocarbons

The concentrations of total aromatic and aliphatic hydrocarbons in dry-aged beef were highest at 14 days of aging compared to 0, 7, and 28 days (*p* < 0.01; Table 4). This suggests that lipid oxidation plays a significant role in the formation of these compounds [22]. Notably, specific hydrocarbons such as dodecane, tridecane, and toluene reached their highest concentrations at 14 days, with hexadecane levels further increasing during the extended aging period from 21 to 28 days. According to Pateiro et al. [27], the main source of hydrocarbons with fewer than 10 carbon atoms was lipid oxidation, whereas those with longer chains are more likely to build up in an animal’s fat, presumably as a result of feeding. The elevated levels of hydrocarbons during the 14-day aging period suggest that this duration may represent a critical phase in the lipid oxidation process, facilitating the formation of volatile hydrocarbons that contribute to the aroma profile of the beef [7].

Among the aromatic hydrocarbons, toluene showed a notable increase at 14 days. Toluene is formed through the pyrolysis of free tyrosine or the oxidative decomposition of unsaturated fatty acids [28]. While typically present in low levels, toluene has been associated with chemical and solvent-like aromas, reflecting its low odor detection threshold and distinct sensory impact [29]. The presence of toluene and other hydrocarbons highlights the complex interplay of biochemical reactions occurring during the aging process, including thermal and auto-oxidative pathways [6]. These findings provide the unique volatile profiles of Thai native beef, which differ from those reported in other beef types, possibly due to breed-specific fat composition, diet, or aging conditions [7]. Further research is needed to explore how these factors influence hydrocarbon profiles and their subsequent impact on the sensory characteristics of aged beef.

### Nitrogen- and oxygen-containing heterocyclic compounds

Nitrogen-containing heterocyclic compounds, such as methyl-pyrazine, and trimethyl-pyrazine, were found to be greater (*p* < 0.01) in dry-aged beef aged for 14, 21, and 28 days compared to dry-aged beef aged for 0 and 7 days (Table 4). Notably, beef dry aged for 14 days had the greatest content of 2,5-dimethyl-pyrazine (*p* < 0.05). These findings suggest that the aging process enhances the Maillard reaction, as longer aging times increase the availability of precursors such as amino acids and reducing sugars that drive the formation of pyrazines [6]. This observation aligns with Ha et al. [30], who reported a progressive increase in pyrazine concentrations with extended aging times across various muscle types. Additionally, the relatively low levels of pyrazines observed in the 0 and 7-day samples may be attributed to fat-derived aldehydes, which can interfere with the Maillard reaction by altering the production of nitrogen-containing heterocyclic compounds [7]. The elevated levels of pyrazines in longer aged beef contribute to a more intense sensory profile characterized by meaty, nutty, and roasted aromas, highlighting their importance in enhancing the flavor quality of aged beef [20].

In contrast, the oxygen-containing heterocyclic compound 2-pentyl furan showed no significant differences across the various aging times (*p* > 0.05; Table 4). Furans are typically derived from amino acid degradation, often under high-temperature conditions, and are associated with sweet, caramel, and beany sensory notes [22]. The absence of variability in 2-pentyl furan concentrations across aging times suggests that the aging process had a limited impact on its formation in this study. However, some authors [31] found that the rancid off-flavor of beef was associated with pentanal and 2-pentyl furan, which has metallic, green, earthy, and beany notes. This study highlights the impact of aging on heterocyclic compounds in Thai native beef, with increased pyrazines enhancing flavor and stable 2-pentyl furan levels. Future research should investigate lipid oxidation, Maillard reactions, and their precursors to further understand their influence on beef flavor and aroma.

### Sulfur-containing compound and ester

Dimethyl disulfide was the sole sulfur-containing compound identified in the dry-aged beef samples (Table 4), with significantly higher concentrations observed after 14 days of aging compared to samples aged for 7, 21, and 28 days (*p* < 0.05). The increased levels of dimethyl disulfide at 14 days suggest a potential influence of microbial activity on the catabolism of sulfur-containing amino acids, such as methionine and cysteine, during the aging process [30]. Dimethyl disulfide is known to be produced through the breakdown of these sulfur-containing amino acids, particularly under the action of bacteria and fungi present in the aging environment [32]. Previous studies, such as those by Sohail et al. [6], reported that longer dry aging periods generally resulted in higher concentrations of sulfur-containing compounds. However, the current study presents a novel finding, with peak levels of dimethyl disulfide occurring at 14 days, followed by a decline in levels at later stages of aging. This is consistent with a prior study by Lee et al. [20], which reported that dry-aged beef from 0 to 14 days had higher concentrations of dimethyl trisulfide compared to samples aged for 21 and 28 days. The decline after 14 days may reflect changes in microbial populations or environmental factors. Further research on microbial dynamics and sulfur metabolism is needed to elucidate their role in shaping beef sensory characteristics.

### Correlation between fatty acids and volatile compounds in cooked dry aged beef

**Figure 1.**
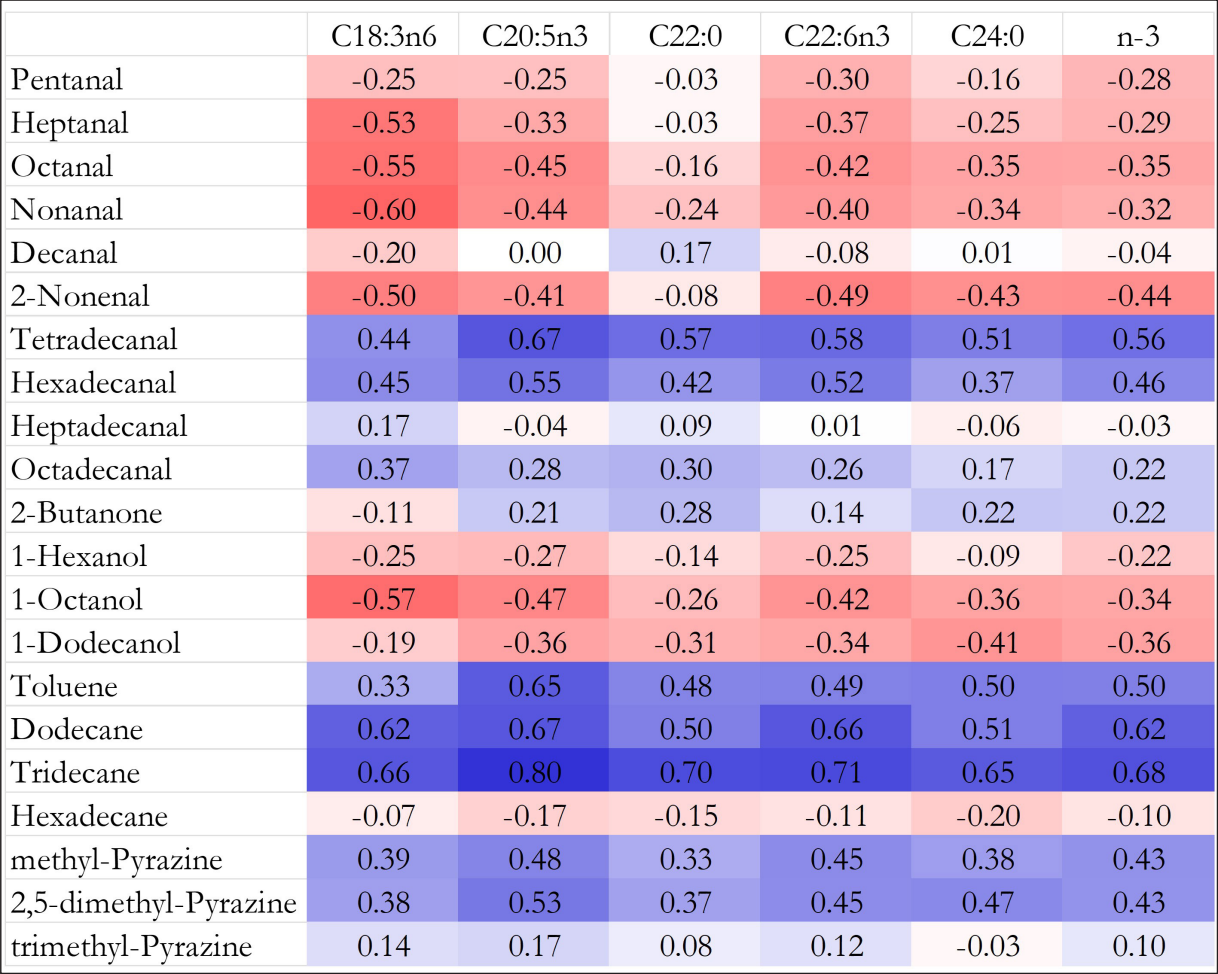
Correlation map of fatty acids and volatile compounds. (Blue and red colors indicate positive and negative correlations, respectively. The darker color showed higher correlation).

The volatile compound profiles in dry-aged beef are closely related to the fatty acid composition, as evidenced by the correlations presented in Figure 1. The oxidation of unsaturated fatty acids, particularly PUFA, plays a key role in the formation of a diverse array of volatile compounds, including aldehydes, ketones, alcohols, aliphatic hydrocarbons, acids, and esters [7]. Our findings align with previous studies [22], showing that long-chain aldehydes, such as tetradecanal and hexadecanal, are positively correlated with the concentration of specific fatty acids. This suggests that the oxidation of PUFA contributes to the formation of these aldehydes, which are known to significantly impact the flavor and aroma profile of cooked meat [23]. Interestingly, our study also observed negative correlations between short-chain aldehydes (C5-C10) and fatty acid concentrations. This may be attributed to the extended aging time, which not only alters the lipid oxidation process but also leads to the accumulation of free amino acids before cooking [6]. These free amino acids could influence the types of volatile compounds produced during the Maillard reaction and lipid oxidation, potentially diminishing the formation of short-chain aldehydes as aging progresses [7].

In addition to aldehydes, we observed positive correlations between two pyrazine compounds formed through the Maillard reaction and the fatty acids [22]. This finding is consistent with the work of Sohail et al. [6], who described how pyrazines are synthesized from the interaction of free amino acids, peptides, reducing sugars, and lipids during cooking. The presence of these pyrazines, known for their roasted, nutty, and meaty aromas, is enhanced by the increasing levels of fatty acids, especially those derived from PUFA [20].

Alcohols exhibited variable correlations with fatty acids. For instance, a negative correlation was noted between 1-octanol and the fatty acids C18:2n6 (linoleic acid) and C20:5n3 (eicosapentaenoic acid). This observation contrasts with the findings of Argemí-Armengol et al. [33], who reported that straight-chain alcohols such as 1-butanol and 1-pentanol can be derived from specific fatty acids like miristoleic acid (C14:1) and linoleic acid (C18:2n6). These discrepancies could reflect differences in the aging time, the oxidation conditions, or the specific enzymatic activities involved in the formation of alcohols from fatty acids [22]. Furthermore, our results showed that branched-chain alcohols, which are typically products of Strecker degradation of amino acids [33], did not correlate significantly with the fatty acid profile in this study. This suggests that the production of branched-chain alcohols might be more influenced by microbial activity or the degradation of specific amino acids during aging, rather than by lipid oxidation processes alone [24].

The positive correlation between aliphatic and aromatic hydrocarbons and fatty acids can be explained by the well-known lipid oxidation pathways [7]. As unsaturated fatty acids undergo oxidation, they produce a variety of hydrocarbons, which contribute to the characteristic aroma of cooked beef [28]. Similarly, the positive correlation between dimethyl trisulfide and fatty acids suggests that this sulfur-containing compound is likely derived from the oxidation of PUFA [34]. These findings align with those of Lee et al. [20], who proposed that variations in sulfur-containing compounds in dry-aged beef may result from differences in the proteolysis of sulfur-containing amino acids, such as methionine and cysteine, during the aging process. In summary, the correlations observed in this study underscore the complex interactions between lipid oxidation, Maillard reactions, and microbial activity during dry aging, which ultimately shape the volatile compound profile and sensory attributes of cooked beef. These findings provide new insights into the role of fatty acids in volatile compound formation and offer a foundation for further research into the mechanisms underlying flavor development in dry-aged Thai native beef.

## Conclusion

The objective meat color and WBSF values decreased as the aging time increased. In terms of chemical composition, the moisture content decreased while the protein and ash content increased with a longer aging time. When the beef was dry-aged for 14 days, there was an increase in the proportion of fatty acids, including C18:3n6, C20:5n3, C22:0, C22:6n3, C24:0, and total n-3 fatty acids. Additionally, with longer aging time, the cooked dry-aged beef had lower levels of short-chain aldehydes (pentanal, heptanal, octanal, nonanal, decanal, and 2-nonenal) and alcohols (1-hexanol and 1-octanol). However, there was an increase in long-chain aldehydes (tetradecanal, hexadecanal, heptadecanal, and octadecanal) with longer aging time. When comparing 14-day dry-aged beef to beef that has not been aged (0 days), there were higher levels of toluene, dodecane, tridecane, methyl-pyrazine, 2,5-dimethyl-pyrazine, trimethyl-pyrazine, and dimethyl trisulfide. These findings indicate that there is a correlation between twelve volatile compounds and six significant fatty acids in the dry-aged beef samples. This suggests that the fatty acid profiles in meat are influenced by aging times and that volatile compounds are associated with the fatty acid profile.

## List of abbreviations

CLA, conjugated linoleic acid; LTL, longissimus thoracis et lumborum; MUFA, monounsaturated fatty acid; PUFA, polyunsaturated fatty acid; SEM, standard error of the mean; SFA, saturated fatty acid; WBSF, Warner-Bratzler shear force.
